# Deep learning classification of systemic sclerosis from multi-site photoplethysmography signals

**DOI:** 10.3389/fphys.2023.1242807

**Published:** 2023-09-13

**Authors:** Sadaf Iqbal, Jaume Bacardit, Bridget Griffiths, John Allen

**Affiliations:** ^1^ Faculty of Medical Sciences, Newcastle University, Newcastle Upon Tyne, United Kingdom; ^2^ Northern Medical Physics and Clinical Engineering, Freeman Hospital, Newcastle Upon Tyne, United Kingdom; ^3^ School of Computing, Newcastle University, Newcastle Upon Tyne, United Kingdom; ^4^ Department of Rheumatology, Freeman Hospital, Newcastle Upon Tyne, United Kingdom; ^5^ Research Centre for Intelligent Healthcare, Coventry University, Coventry, United Kingdom

**Keywords:** deep learning, machine learning, photoplethysmography, pulse, Raynaud’s, scleroderma, systemic sclerosis

## Abstract

**Introduction:** A pilot study assessing a novel approach to identify patients with Systemic Sclerosis (SSc) using deep learning analysis of multi-site photoplethysmography (PPG) waveforms (“DL-PPG”).

**Methods:** PPG recordings having baseline, unilateral arm pressure cuff occlusion and reactive hyperaemia flush phases from 6 body sites were studied in 51 Controls and 20 SSc patients. RGB scalogram images were obtained from the PPG, using the continuous wavelet transform (CWT). 2 different pre-trained convolutional neural networks (CNNs, namely, GoogLeNet and EfficientNetB0) were trained to classify the SSc and Control groups, evaluating their performance using 10-fold stratified cross validation (CV). Their classification performance (i.e., accuracy, sensitivity, and specificity, with 95% confidence intervals) was also compared to traditional machine learning (ML), i.e., Linear Discriminant Analysis (LDA) and K-Nearest Neighbour (KNN).

**Results:** On a participant basis DL-PPG accuracy, sensitivity and specificity for GoogLeNet were 83.1 (72.3–90.9), 75.0 (50.9–91.3) and 86.3 (73.7–94.3)% respectively, and for EfficientNetB0 were 87.3 (77.2–94.0), 80.0 (56.3–94.3) and 90.1 (78.6–96.7)%. The corresponding results for ML classification using LDA were 66.2 (53.9–77.0), 65.0 (40.8–84.6) and 66.7 (52.1–79.2)% respectively, and for KNN were 76.1 (64.5–85.4), 40.0 (19.1–63.9), and 90.2 (78.6–96.7)% respectively.

**Discussion:** This study shows the potential of DL-PPG classification using CNNs to detect SSc. EfficientNetB0 gave an overall improved performance compared to GoogLeNet, with both CNNs performing better than the traditional ML methods tested. Our automatic AI approach, using transfer learning, could offer significant benefits for SSc diagnostics in a variety of clinical settings where low-cost portable and easy-to-use diagnostics can be beneficial.

## 1 Introduction

### 1.1 Background

Systemic Sclerosis (SSc, aka Scleroderma) is a complex, rare, connective tissue disease (CTD) involving the collagen, major organs, the blood vessels and the immune system (van den Hoogen et al., 2013; Di Battista et al., 2021), in which extensive fibrosis and vascular alterations take place. It has significant morbidity and mortality (van den Hoogen et al., 2013), and in the UK an estimated prevalence of 307 per million (95% CI: 290–323), with the highest occurrence in the 70–84 years age group (Royle et al., 2018). SSc (the two most common variants are limited cutaneous variant (lcSSc), and diffuse cutaneous (dcSSc)) is often associated with Raynaud’s phenomenon (RP), a condition in which recurrent, reversible vasospasm of the digital small arteries, arterioles, pre-capillary and post-capillary venules occurs on exposure to cold or emotional stress (Hughes and Herrick, 2016; Silva et al., 2016). RP is common and in the UK is reported to affect up to 10 million people (SRUK, 2023). About 1 in 16 women and 1 in 50 men with Raynaud’s develop SSc, usually between the ages of 25 and 55 (NHSinform, 2023; Haque, 2020; Belch, 2017). It is usually sub-categorised into: a) Primary RP (PRP) when no underlying cause condition is known (idiopathic); b) Secondary RP when RP is linked to an underlying disease such as SSc or dermatomyositis or to the intake of certain drugs. Secondary RP is typically seen in approximately 90%–96% of patents with SSc and often precedes the development of SSc by an average time of 10.4 years (Spencer-Green, 1998; Pauling et al., 2019). Clinical specialists differentiate secondary RP from PRP by checking for symptoms associated with secondary RP such as the age at onset (secondary RP is usually after 30 years of age), detecting abnormal immunology e.g., certain autoantibodies, observing nailfold capillaries, ulceration of digits, checking for fibrosis in the lungs or other organs, and skin thickening which is the hallmark of SSc. However, diagnosing SSc is not always easy as its symptoms resemble other conditions such as PRP and early symptoms of diseases such as systemic lupus erythematous (where 10% and 45% of patients show Raynaud’s phenomenon). Early detection and management of the disease is a must, to improve the morbidity and mortality in patients (Walker et al., 2007). This in turn requires a multi-disciplinary and collaborative effort involving clinical specialists and testing. It can take more than one visit to an expert Rheumatology specialist to diagnose the disease, especially in the early stages. Identification of internal organ involvement and its severity is also important.

### 1.2 Current methods of SSc diagnosis

These involve extensive and costly testing for autoantibodies and markers of organ involvement. Sometimes, it is difficult to distinguish between SSc and non-SSc cases as patients have overlap conditions. Nailfold capillaroscopy (NFC) is another key technique used to help diagnose SSc. NFC is a non-invasive, optical imaging technique (Allen and Howell, 2014; Eriksson et al., 2014) that is used by an expert operator to visually inspect the microcirculation in the nailfold capillaries of the distal papillae and hence assess pathological/morphological changes associated with SSc such as capillary “dilatation”, distribution and density (for “drop-out”), bushing (for “angiogenesis”) and microhaemorrhage (extravasation). Tests such as NFC, however, are usually currently performed in specialist hospitals and are not at all readily available to all patients.

There is huge scope to look for alternative, low-cost technologies to assess SSc. Photoplethysmography (PPG) is one such technique, which is non-invasive and optically assesses the circulation. The working principle of a PPG-based system uses a suitable light source such as infrared or near-infrared light to study the heart-synchronous changes in blood volume in the microvascular bed of tissues such as skin (Allen, 2007; Elgendi, 2012; Kyriacou and Allen, 2021). Additional key advantages of PPG are its portability and its versatility to be used in a range of settings such as measurement labs as well as ambulatory assessments (wearable sensors, Charlton et al., 2023). PPG is currently widely used in different clinical applications (Johnson et al., 2020) including for pulse oxygen saturation measurement (SpO2) (Ma et al., 2018), cardiovascular health (heart rate, blood pressure, blood vessel and arterial stiffness) monitoring (Castaneda et al., 2018), and for studying hypertension (Liang et al., 2018).

### 1.3 Recent works

The potential of PPG for detecting patients with SSc has already been explored using conventional optical pulse wave analysis techniques but the literature here appears to be limited to date. The largest SSc PPG study reported to date is by Rosato et al. (2010) with 105 SSc (compared to 96 PRP and 85 healthy controls) using a Termoflow type PPG instrument. The authors found that the mean amplitude of the PPG sphygmic wave was significantly lower in the PRP group than in the SSc group (mean ± standard deviation = 11 ± 10, given in arbitrary units (a.u.) vs. 24 ± 24 a.u. for SSc). The mean amplitude was also significantly lower in SSc than in HC (56 ± 19 a.u. for HC). A further study by Rosato et al. (2011) using bilateral PPG measurements found a homogeneous pattern (meaning uniformity of morphology and amplitude of sphygmic PPG wave across all 10 fingers) for 95% of the HCs and 93% of the PRPs but was only 28% for the SSc group. McKay et al. (2014) investigated multi-site PPG in 19 SSc, 19 PRP and 23 HC by studying measures of arterial, endothelial and peripheral autonomic dysfunction under a dynamic 3 phase testing protocol. The authors found that measures attributed to endothelial function were significantly impaired in SSc (*p* < 0.02), but with no difference between the HCs and PRPs. The authors reported that the Receiver Operating Characteristic (ROC) based classification accuracy was 81% (sensitivity 90%, specificity 74%) for separating SSc from HCs, and 82% (sensitivity 84%, specificity 79%) for separating SSc from PRPs. Mamontov et al. (2020) employed imaging PPG (iPPG) and studied 19 SSc and 21 HC participants (with age- and sex-matched balanced classes) and from the pulse arrival time (PAT) showed a significant increase in its variability in SSc patients as compared to HCs (52 ± 47 ms vs. 24 ± 13 ms, *p* = 0.01). These earlier works show the potential of PPG as a tool to help investigate SSc but such approaches from the literature have relied on extracting a range of pulse features based on domain knowledge, and then performing specific feature selection to try and improve classification performance. To the authors’ best knowledge, there are no papers on contact type PPG measurements using analysis based on deep learning (DL) for the assessment of SSc patients. The aim of this pilot study was to utilise DL in a novel way by applying it to automatically identify the presence (or absence) of SSc from multi-site PPG measurements collected under a dynamic 3-phase test protocol.

## 2 Materials and methods

### 2.1 Study participants

Consecutive patients were approached by specific autoimmune connective tissue disease rheumatologists or the connective tissue disease nurse specialist from the Rheumatology outpatient population attending Freeman Hospital, Newcastle upon Tyne. The SSc participants were each diagnosed by an expert consultant Rheumatologist at Freeman Hospital, using the 1980 American College of Rheumatology (ACR) preliminary criteria for the classification of systemic sclerosis (Subcommittee for Scleroderma Criteria, 1980). PRP patients were diagnosed by the same clinical team, as having vasospastic symptoms for >2 years, with routine practice assessments and no other underlying medical or mechanical cause. SSc and PRP participants were recruited from the Rheumatology outpatient clinic at Freeman Hospital, Newcastle upon Tyne. Healthy Control (HC) participants were recruited from volunteers - largely from the University of the Third Age (U3A, Wearside Branch), and from staff and students of Newcastle Hospitals and Newcastle University. The HC participants had no known underlying health condition (e.g., diabetes mellitus and hypertension). For each patient a range of clinical and demographic data were collected for the study, including participants’ pertinent medical history, and the tests done in the hospital to assess and diagnose SSc patients. All participants were permitted to continue their regular medication, including vasodilator treatment, at the time of their recruitment and informed consent was taken. Ethics approval for the original study data collection was granted by the National Research Ethics Service (NRES) Committee Northeast (County Durham and Tees Valley 1 REC, 07/H0905/72 2008). Ethics permission for re-analysis of the anonymised PPG waveform data for Sadaf Iqbal’s PhD studentship project was obtained from Newcastle University (Reference 7273/2018, with an extension to the study 17138/2021).

### 2.2 Multi-site PPG measurements and pre-processing

**FIGURE 1:**
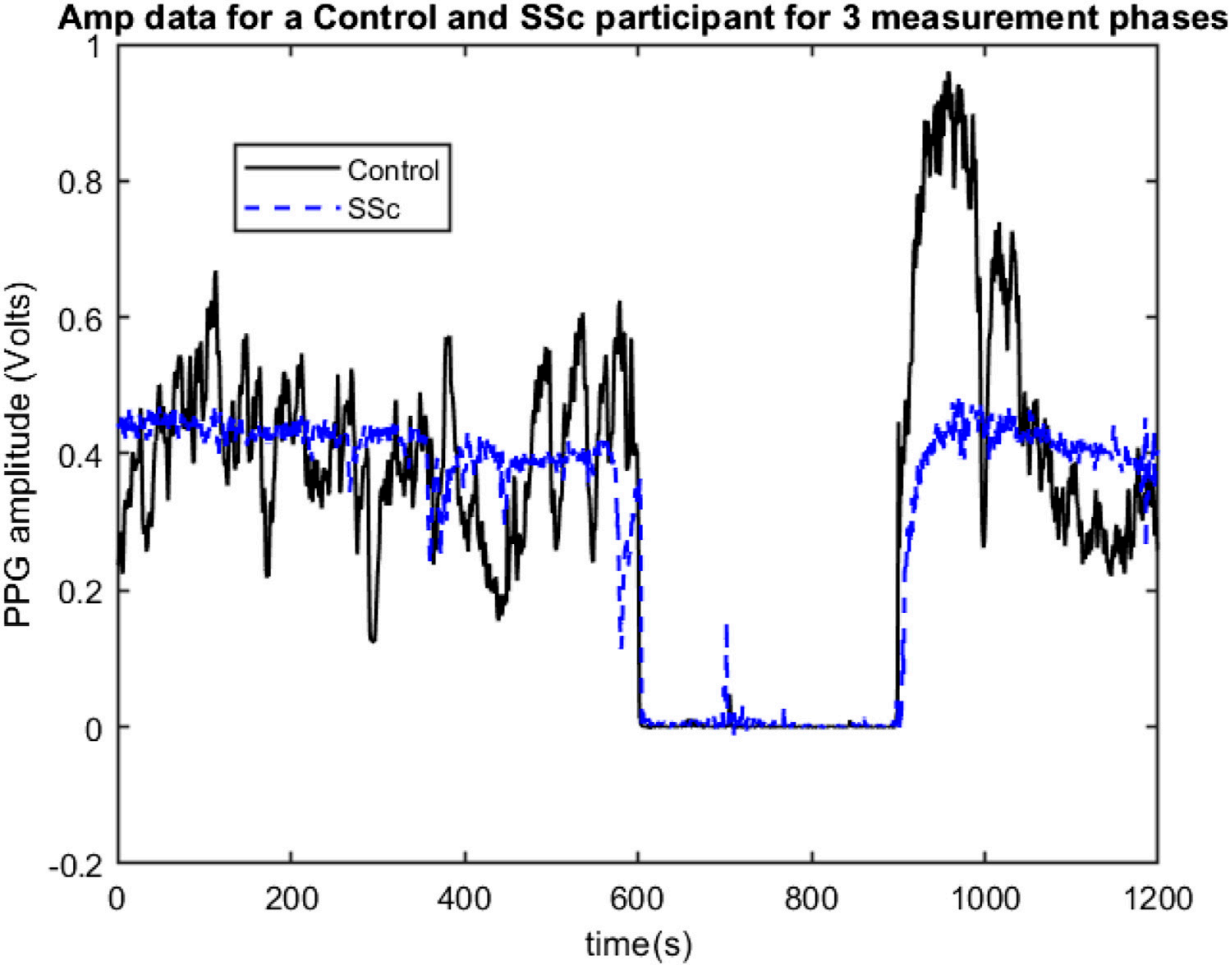
Example 3-phase PPG Amp data recordings for a Control (black line) and for a SSc participant (Blue line) showing beat-to-beat amplitude changes for the left finger site, with 0–600 s = Baseline phase, 600–900 s = Occlusion phase, and 600–1200 s = Flush phase.

The PPG data set measurements were originally collected by Dr Neil McKay, Rheumatologist, between 2009 and 2011 at Freeman Hospital’s microvascular research facility, with 3-phase measurement protocol developed and physiological measurement training supported by expert PPG operator JA. Participants were firstly asked to lay comfortably in a symmetrical supine position for a period of at least 15 min whilst acclimatising in a warm normothermic temperature-controlled (23°C ± 1°C) clinical measurement room. Multi-site PPG waveforms were then collected simultaneously for 20 min from 6 symmetrical, body sites namely: right and left earlobes, index finger pads and great toe pads respectively, using optically and electronically matched amplifiers (bandwidth 0.5–20 Hz) and captured to computer at a sampling frequency (Fs) of 2000 Hz. The 3 phases of measurement were: subject resting supine (10 min, Baseline phase); an arm pressure cuff inflated at 300 mmHg to stop the arterial blood flow into the left arm (for 5 min, Occlusion phase); then at 15 min the cuff pressure was quickly but carefully released, and the degree of reactive hyperaemia monitored for a further 5 min (Flush phase). Figure 1 shows examples of 3-phase PPG beat-to-beat amplitude data for the left finger measurement site of a Control participant and a SSc participant.

**FIGURE 2:**
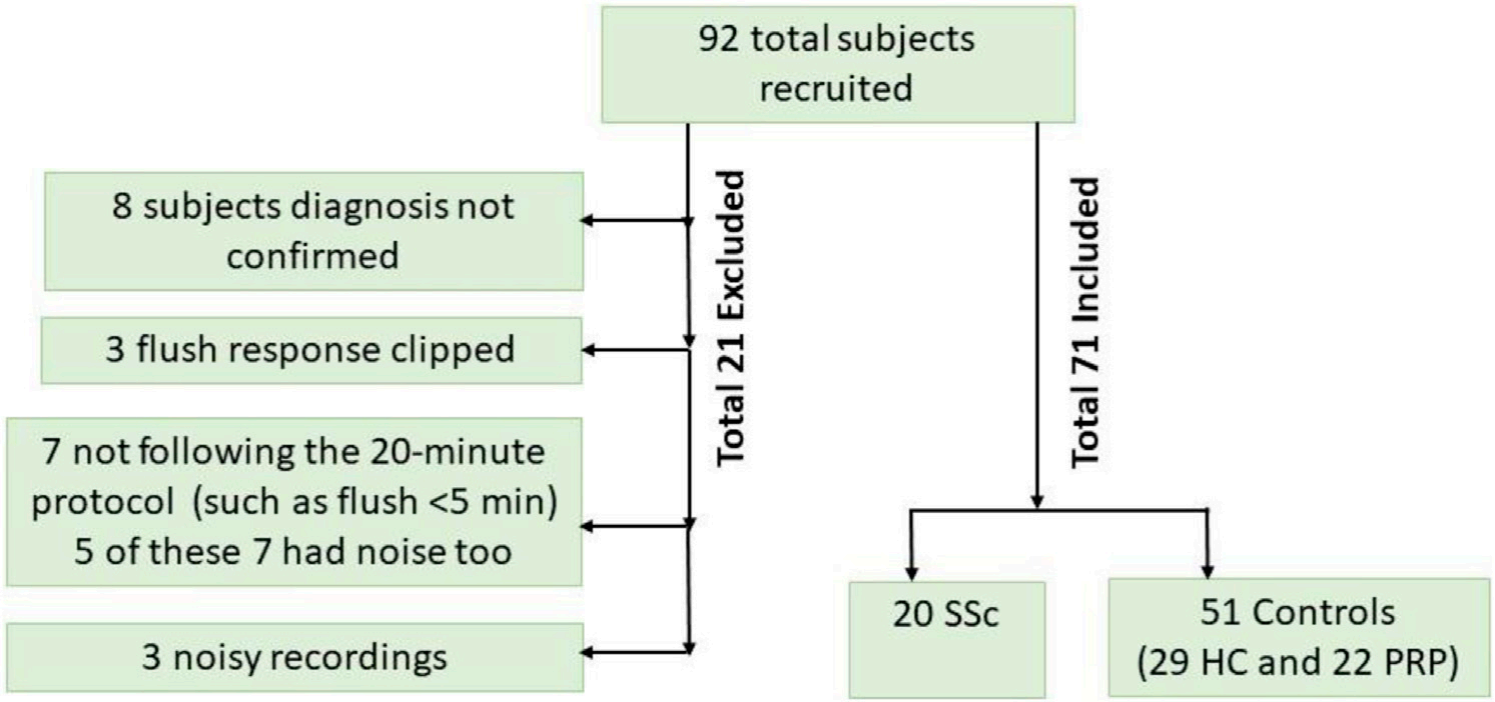
Participant flowchart showing included and reasons for the excluded subjects.

For this study involving advanced analysis of the data, a visual analysis of the PPG data was carried out by operator SI to check for the presence of unexpected artifacts (such as that caused by unreliable PPG probe skin contact or when a study did not follow protocol) or a distorted flush response was evident. Some of the 92 participants originally entered into the study had to be excluded: original PPG data collected using too high a manual gain setting causing a high flush which saturated i.e., electronically clipping and making unusable a PPG trace for the left study arm (N = 3); participants not following the 20-min protocol (N = 7). In total, PPG and ECG data from a total of 20 SSc, 22 PRP and 29 HC participants were included and analysed using the techniques described in the next section. Figure 2 shows a participant flowchart summary for the included and excluded subjects in our pilot study.

Healthy controls and patient controls (i.e., PRP) participants were combined in a single control group as they both belong to clinical class of non-life-threatening conditions as opposed to SSc group which needs clinical attention and early identification and management. Also, noting here that although pulse amplitudes can be different between the groups and from the same subject if tested on different days, the literature suggests that PPG pulse measures are similar across the two groups. The study by Rosato et al. (2011) had shown that PPG shows a homogeneous pattern, i.e., a uniformity in morphology as well as amplitude of PPG sphygmic waves across the fingers, in 95% of the healthy control subjects and 93% of the PRP patients as opposed to this being present in only 28% of the SSc patients. McKay et al. (2014) also had found no differences between healthy controls and PRP for the case of dynamic physiological testing i.e., the derived PPG median flush response slope and flush response value.

In this study a PPG signal was pre-processed with only a normalisation stage, with each of the 6 PPG signals divided by their respective PPG amplifier channel gain setting. There was no additional signal filtering performed by the computer.

### 2.3 Deep learning classification

This data analysis was carried out on a 64-bit Windows 10 and 14 Core PC fitted with a single NVIDIA GeForce RTX 2080 Ti GPU card which used MATLAB software. Here, the PPG time series was converted into scalogram images for the study subjects using continuous wavelet transform (CWT). Scalograms give a time-frequency (T-F) image representing the percentage of signal energy contained in different frequency bands (Gandhi et al., 2014), with the *x*-axis representing time and *y*-axis representing frequency and a signal varying in colour intensity over the T-F plane.

**FIGURE 3:**
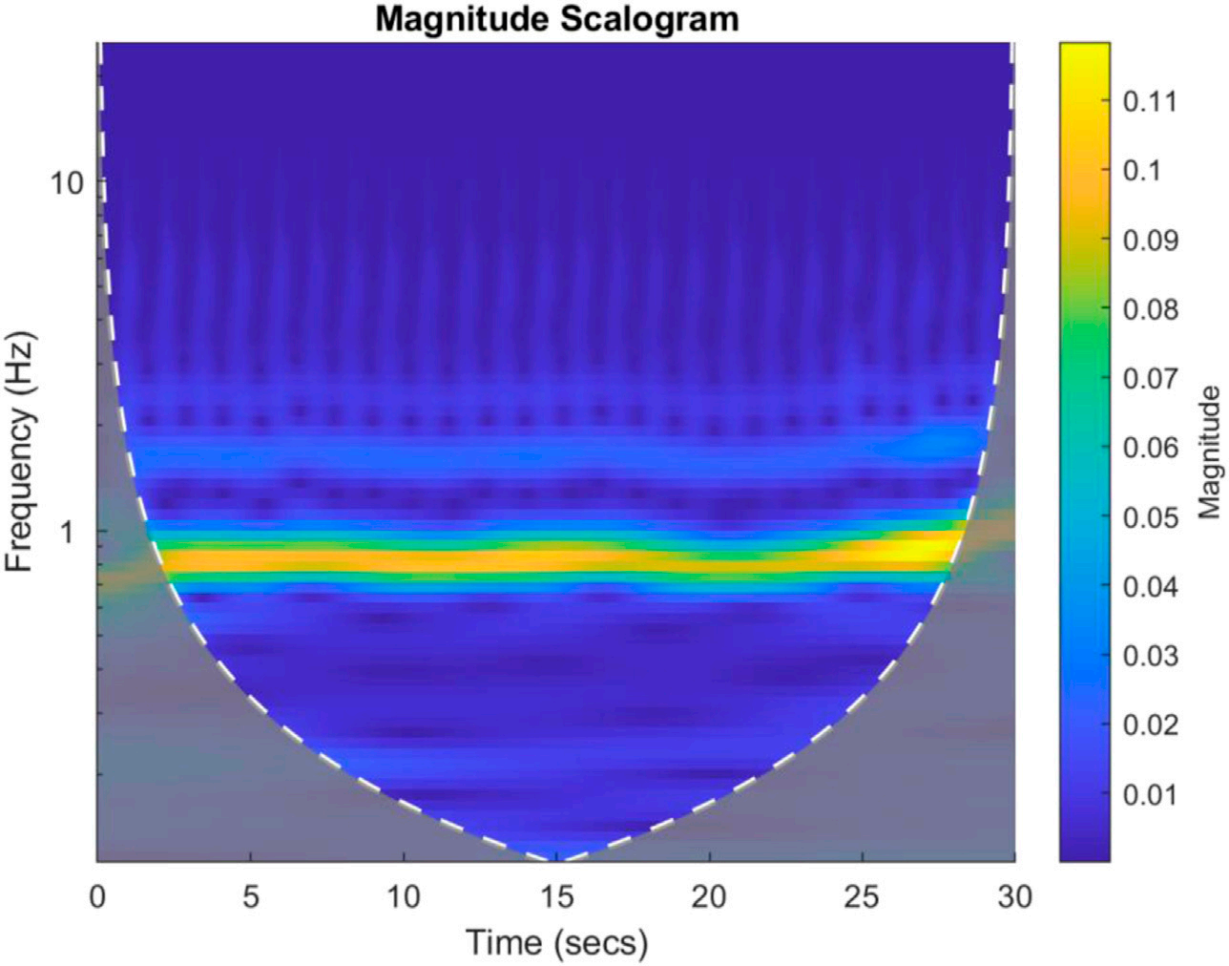
An example 30 s scalogram from a Control subject. The magnitude of the scalogram is shown in the colour bar to the right of the image. The brighter colours represent higher signal energy at a particular frequency.

Each PPG channel’s gain normalised time series was divided into consecutive 30 s non-overlapping windows and for each 1D time window, a 2D scalogram image along with its label was then generated using continuous wavelet transform (CWT) methods. The Morse mother wavelet was used in CWT as this type of analytic wavelet is very useful to analyse signals with time-varying amplitude and frequency (PPG here) (Wachowiak et al., 2018) and Voices per Octave was selected as 12 to keep the computational complexity low (Wachowiak et al., 2018). The 30 s period for plotting scalograms was selected based on initial exploratory scalogram plotting to select meaningful T-F resolution and hence extract meaningful features from the scalogram images. Noting that with the uncertainty principle, the greater length of time window means that frequency resolution is higher but time resolution is lower, and *vice versa*. Hence, for the data under consideration a 30 s epoch provided a reasonable trade-off between the time and frequency resolutions. Figure 3 shows a 30 s sample scalogram with frequencies ranging from 0–20 Hz (noting each PPG was already bandpass filtered in this range using analogue electronics during physiological data acquisition). The scalogram was calculated using Eqs 1, 2 below:

$$S=\left|coef.*coef\right|where\;coef\;are\;the\;CWT\;coefficients\;over\;a\;time\;window$$ (1)

$$Scalogram=\left(100\times S\right)/\left(\sum S\right)$$ (2)

**FIGURE 4:**
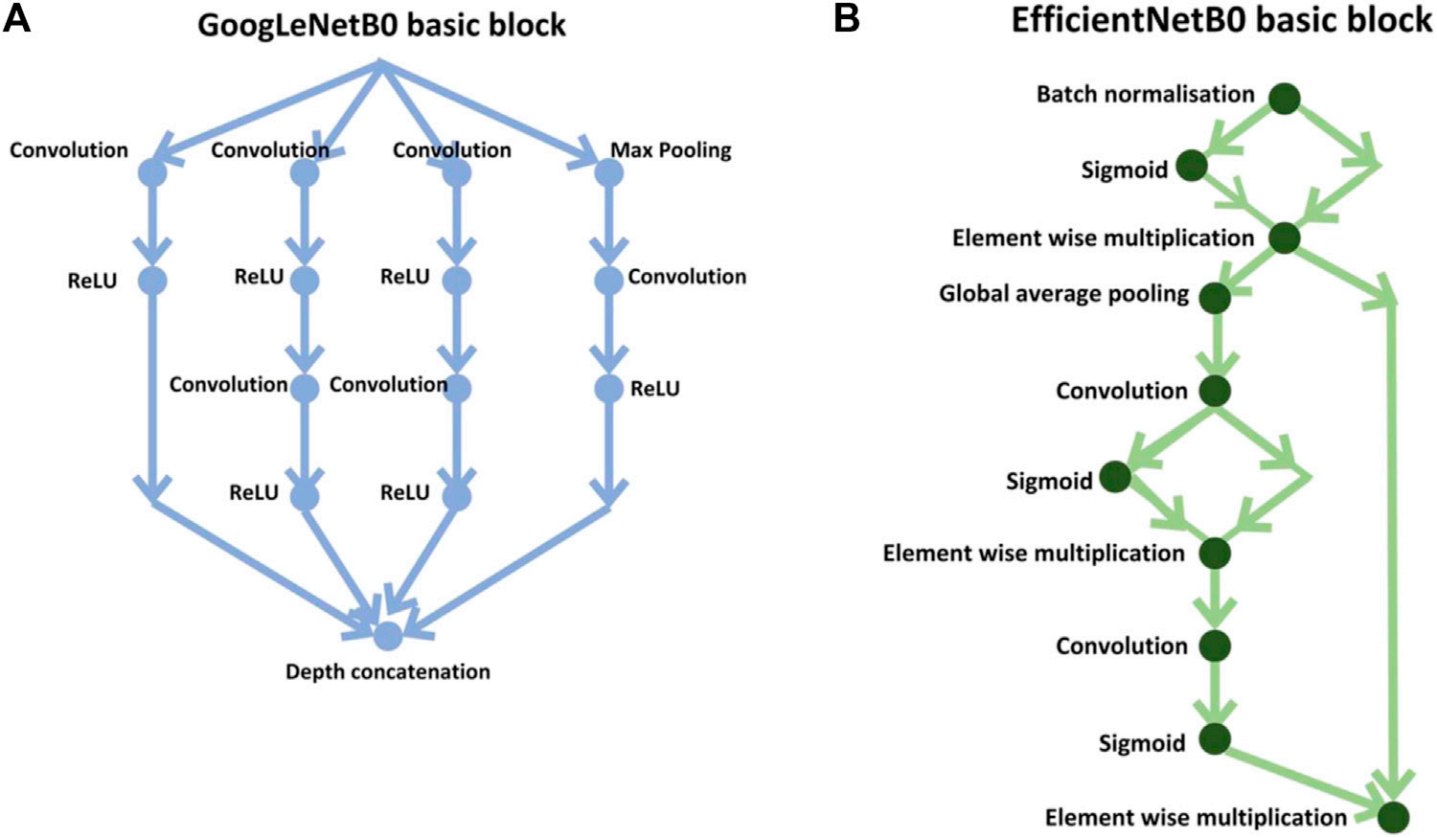
Basic building blocks of GoogLeNet and EfficientNetB0 are shown in diagrams **(A,B)**, respectively. The small nodes represent a layer each specified by its name.

**FIGURE 5:**
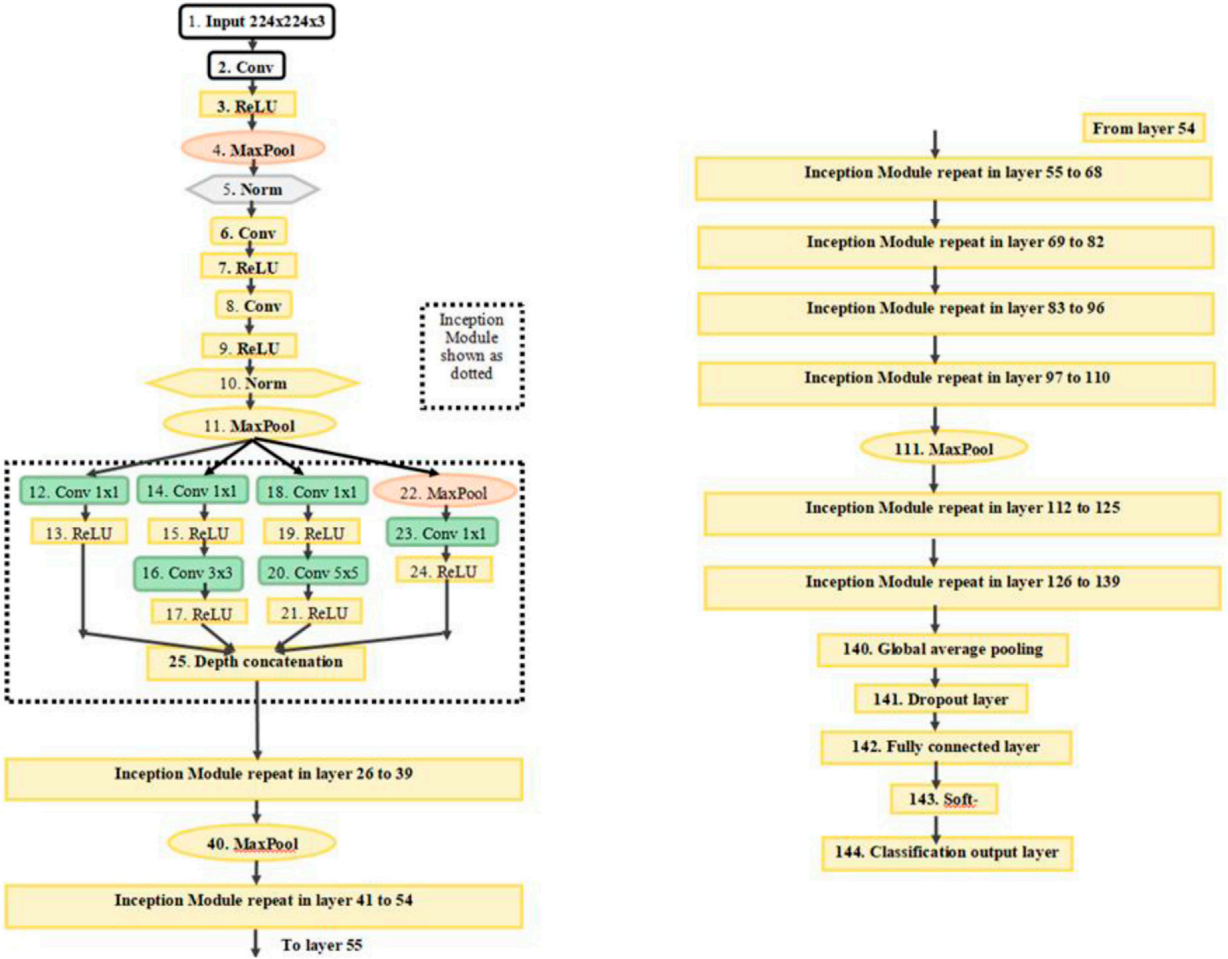
The architecture of GoogLeNet. Conv, convolution; Norm, normalization; MaxPool, maximum pooling. The inception modules repeat but filter depth is different in different modules (Szegedy et al., 2015).

**FIGURE 6:**
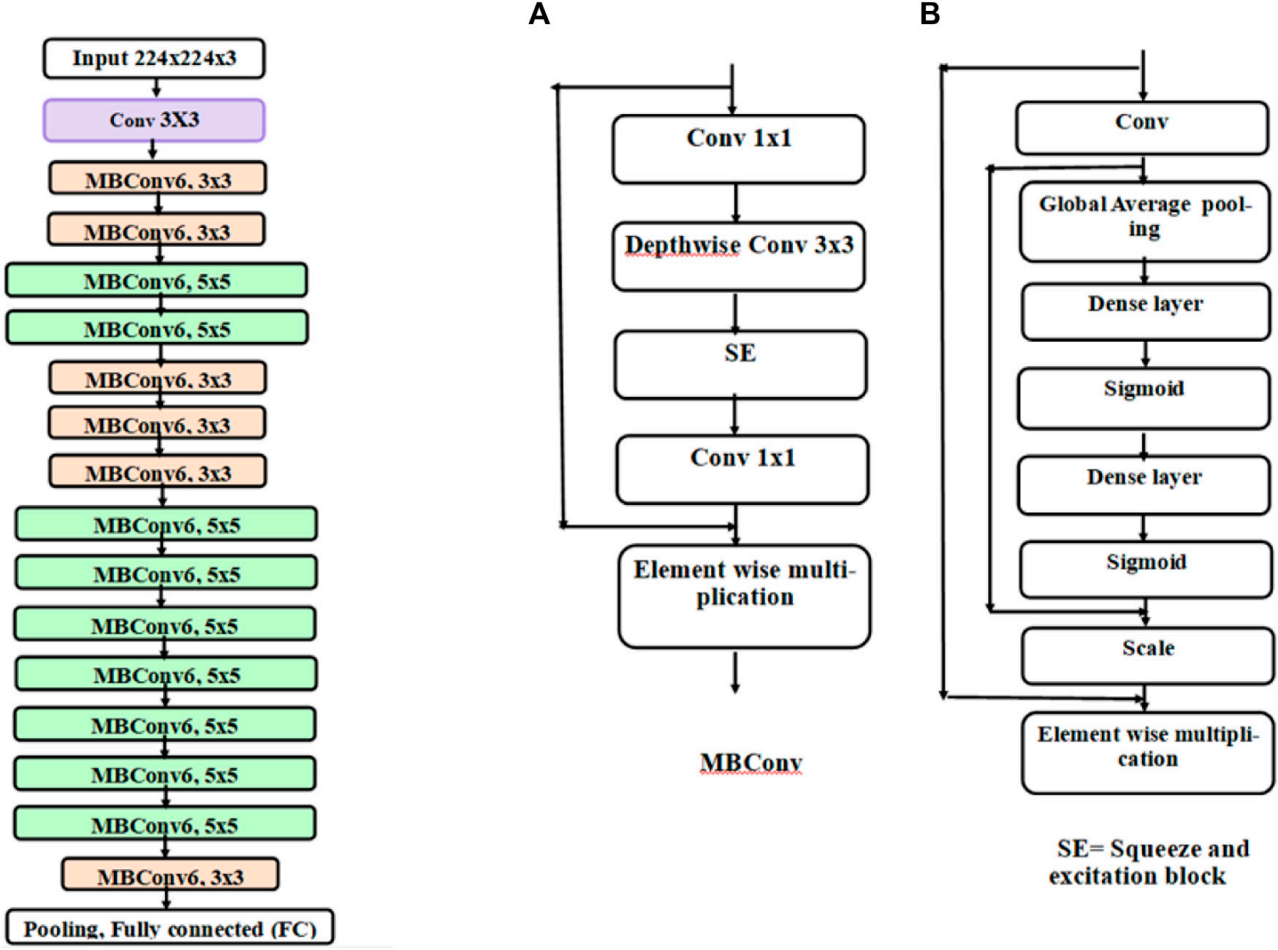
Architecture of EfficientNetB0. Each block represented by different colours is made up of different layers. The basic block of EfficientNetB0 is an inverted mobile bottleneck (MBConv) and its structure is shown in diagram **(A)** and the structure of squeeze and excitation (SE) block is shown in diagram **(B)**

In the first stage, the results were calculated using the number of images that were classified into SSc *versus* Control (‘image-based performance’). Two pretrained convolutional neural networks (CNN) from MATLAB were used (GoogLeNet (Szegedy et al., 2015) and EfficientNetB0 (Tan et al., 2019)) to learn the PPG T-F features and then perform the classification. These networks have already been previously trained on millions of high-resolution images from the ImageNet database (Krizhevsky et al., 2017). GoogLeNet (structure comprising 144 layers) was the winner of 2014 ImageNet competition and had least number of parameters (∼6.9 million) as compared to other pretrained models available in MATLAB at the time of study. EfficientNetB0 (290 layers) which was introduced in 2019 (parameters ∼5.3 million) represents a newer generation of CNNs, based on ResNet design, and had been designed to work better and faster than the hitherto available CNNs (Tan and Le, 2019), giving 2 key types of CNN to implement and explore respective performances. Figure 4 shows the basic building blocks for these two types of CNN network. Figures 5, 6 respectively show the network architectures of GoogLeNet (Szegedy et al., 2015) and EfficientNetB0 (Tan et al., 2019; Tan and Le, 2019).

For the PPG dataset, 16,188 labelled scalogram images were generated for the 71 participants covering all 6 PPG sites, i.e., channels of the multi-site PPG system. The scalogram images were rescaled to 224 × 224 × 3 size to match input dimensions of GoogLeNet and EfficientNetB0 using a standard MATLAB function “imresize” which applies a simple scale transformation to the original image using bicubic interpolation which is a standard algorithm used in image resizing (Hashemi et al., 2015). Data were divided into 10 mini batches (batch size 1620 images) to reduce computational time. The learning rate was 0.005. Optimisation utilised the stochastic gradient descent method (Tian et al., 2023) as this is computationally faster and can converge quicker than other optimisation algorithms. The loss function used was cross-entropy loss. Ten-fold stratified cross validation was carried out on 71 participants wherein 9/10 of the participants’ images were used in training and 1 mutually exclusive (1/10 of the participants’ images) were used for its testing, this process was repeated 10 times. The image wise combined confusion matrix obtained after 10-fold CV was used to calculate measures of classification performance. The two networks train on the input training data and adapt their weights, to learn the features of the data. The approximate training time in any fold of training was about 240–300 min. The approximate testing time for any test fold was between 20–30 min.

**FIGURE 7:**
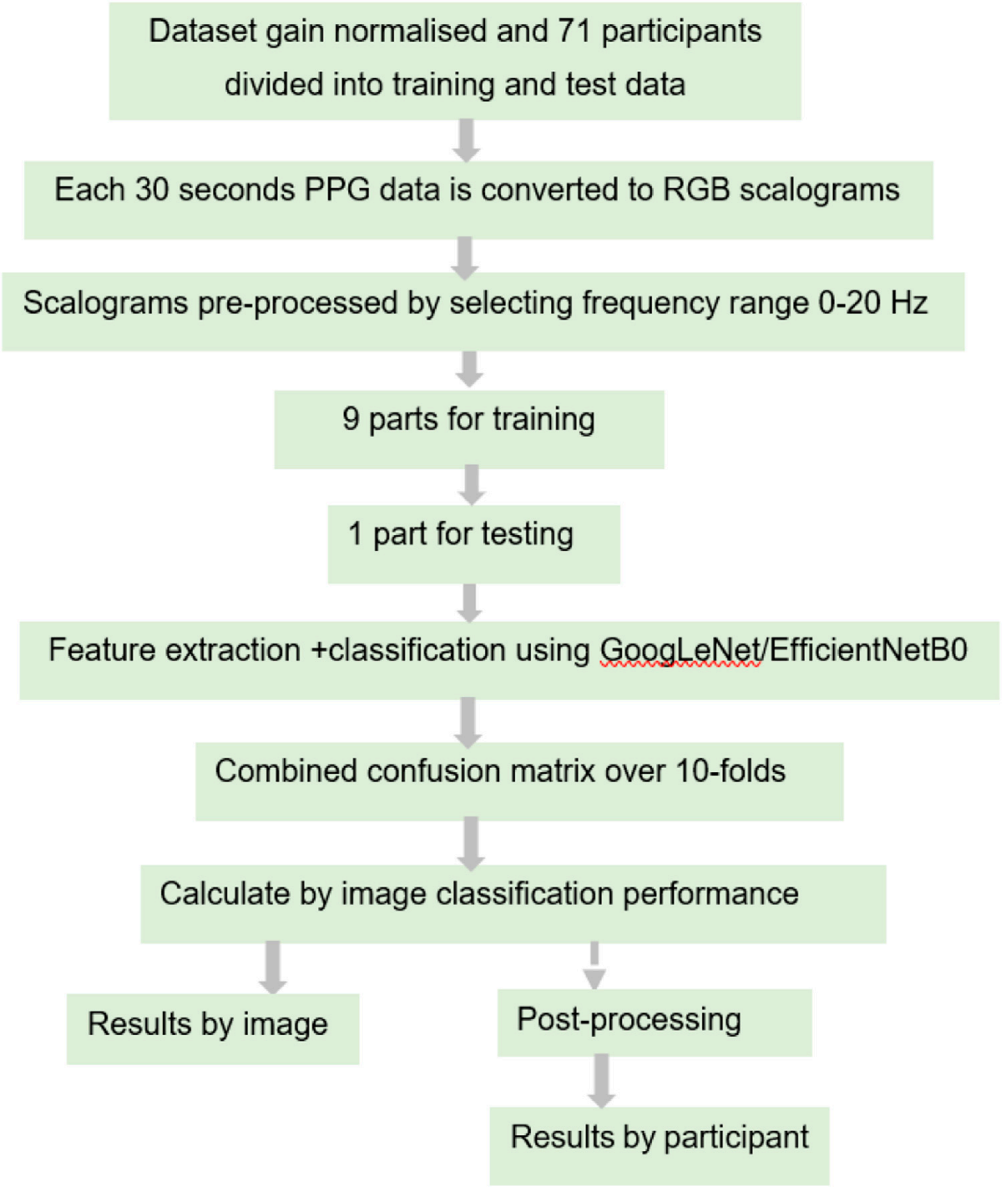
Block diagram of the steps and methodology for the DL analysis.

The SSc *versus* Control classification was performed based on number of images and this is called as image-based classification throughout. A post-processing step was also applied which calculated the number of images classified in each category per each individual test participant, to provide SSc *versus* Control classification based on number of participants. In this case, the majority class of images in each test participant was considered as the class of the output label. This was compared against the ground truth label (i.e., SSc diagnosis) which was clinically determined beforehand. The steps and methodology of the DL analysis are summarised in the block diagram in Figure 7.

### 2.4 Comparator ML method using wavelet time-frequency classification

This is a comparator ML method using the discrete wavelet transform (DWT) to compare with the performance of the T-F based DL classification. DWT method has advantages over other T-F methods such as CWT such in being computationally faster and the ability to analyse the input signal into desired constituent frequency bands. The same participant-wise partitions of the training/test data sets were used as for the DL classification work to allow a fair inter-comparison between methods.

Ten-level DWT decomposition (Mohamed and Deriche, 2014) was carried out per channel PPG using Daubechies 4 (db4) mother wavelet thereby producing 10 levels of detailed coefficients (d1, d2, ……. , d10) and approximate coefficients (shortened as ap in this work) for each channel. The mother wavelet db4 was chosen as it matches the shape of the PPG pulse more than any other wavelet, and hence is the best choice to calculate the wavelet transform of the signal. Since the PPGs had already been bandpass filtered (i.e., 0.5–20 Hz) at data acquisition then only the detailed coefficients containing these relevant frequencies namely, d6 (frequency range 15.63–31.25 Hz), d7 (7.81–15.63 Hz), d8 (3.91–7.81 Hz), d9 (1.95–3.91 Hz), d10 (0.98–1.95 Hz) and ap (0.49–0.98 Hz) were selected for further analysis. To make the computational complexity less and thus the algorithm faster, 4 features were extracted from each of the 6 frequency bands for each channel, thereby giving 6*6*4 = 144 features per participant. PPG features extracted were Energy, Entropy, Mean absolute value and Skewness, as defined in Eqs 3–5.

**Energy**: Energy of the PPG DWT coefficients is calculated as the sum of the squares of all the sampled amplitudes for a single channel and is defined by Eq. 3:

$$Energy=\sum_{i=0}^{N}x_{i}^{2}$$ (3)

Where x_i_, the i^th^ data instance and N are the total number of sample values.

**Entropy**: Entropy describes the irregularity, complexity, or unpredictability characteristics of a signal. In this work entropy was used as a feature to quantify the irregularity of the PPG time series and hence Shannon entropy was calculated as given by Eq. 4:

$$Entropy=\sum_{i=1}^{N}\left\{x_{i}\;*\;\log_{2}\left(1/x_{i}\right)\right\}$$ (4)

**Mean absolute value**: The absolute value of the average of each channel within the data matrix.

**Skewness**: Skewness can be used as a measure to describe the asymmetry around the mean of the data sample. If the value of skewness is less than zero, the data has more spread around the left-hand side of the mean, if greater than zero, it is more towards the right-hand side of the mean and if equals to zero, the data can be considered as symmetrically distributed. For a dataset, skewness can be described by Eq. 5:

$$Skewness=\frac{E{\left(x_{i}-\mu \right)}^{3}}{\sigma^{3}}$$ (5)

Where µ is the mean of x, σ is the standard deviation of x, and E(t) represents the expected value of the quantity t.

The extracted features were then fed into the linear discriminant analysis (LDA) and into the K-Nearest Neighbour (KNN, set at K = 9) analysis to classify SSc *versus* Control.

### 2.5 Statistical analysis

Demographic data were expressed using mean (±standard deviation, SD) values. Since the subjects are grouped into 2 distinct SSc and Control classes with each group having independent and different participants, hence unpaired Student’s t-test was used to study the mean values of SSc and Control groups. A *p*-value <0.05 was the level of statistical significance. A crosshair plot illustrating a meta-analysis overview of classifier performance (test sensitivity *versus* False Positive Rate i.e., 1-specificity) was produced using the *mada* command from R (RStudio version 1.4.1106).

## 3 Results

Table 1 overviews the demographic details for the study participants. Clearly, there were more females than males in the study (18 out of 20 in the SSc group and 45 of 51 in the Control group), but with no significant difference between the groups (*p* = 0.83). Noting, this is representative as SSc is more prevalent in females than males (van den Hoogen et al., 2013). There was a significant but modest difference for age (*p* = 0.007) with SSc [62 (18) years] older than Controls [50 (11) years] overall. There was a marginally higher BMI for the SSc patient group [26.4 (4.1) kg/m^2^] compared to Controls [23.9 (3.9) kg/m^2^ (*p* = 0.020). There was no significant difference found for SBP (*p* = 0.712) and borderline significance for the DBP (*p* = 0.050) between the groups.

**TABLE 1:** Demographic details of the study participants.

Key demographics, with mean (SD values)						
Mean ± SD	Sex	Age (years)	BMI (kg/m^2^)	SBP (mmHg)	DBP (mmHg)	Immunology: Specific SSc autoantibodies
HC	23F, 6M	47 (18)	24.1 (3.8)	135 (21)	84 (6)	All presumed negative
PRP	22F, 0M	54 (16)	23.7 (4.0)	138 (16)	84 (7)	All negative
All Controls (HC + PRP)	45F, 6M	50 (18)	23.9 (3.9)	136 (19)	84 (7)	All negative
SSc	18F, 2M	62 (11)	26.4 (4.1)	134 (24)	80 (9)	10 positive
*p*-value between SSc and Control	Proportion of males between the classes, *p* = 0.83	**0.007**	**0.020**	0.712	0.050	

SD, standard deviation; BMI, body mass index, D/SBP, diastolic/systolic blood pressure, M = male, F = female. Note: the bold numbers in *p* values represent statistically significant levels between Controls and SSc.

The key diagnostic test related results (in %) and the respective 95% CI ranges for image based and participant based SSc *versus* Control classification analyses are summarised in Tables 2–5. For image-based classification the diagnostic test accuracy for GoogLeNet was 83.2 (95% CI range 82.6–83.8) %, sensitivity 73.7 (72.4–74.9) and specificity 86.9 (86.3–87.6). Performance was improved for EfficientNetB0 with 88.4 (87.8–88.8) %, 80.4 (79.2–81.5) and 91.5 (90.9–91.9), respectively. For participant-based classification the diagnostic test accuracy for GoogLeNet was 83.1 (72.3–90.9) %, sensitivity 75.0 (50.9–91.3) and specificity 86.3 (73.7–94.3), and once again the performance was improved for EfficientNetB0 with 87.3 (77.2–94.0) %, 80.0 (56.3–94.3), and 90.1 (78.6–96.7), respectively.

**TABLE 2:** Combined confusion matrix obtained in this analysis for number of image-based classification for GoogLeNet and EfficientNetB0 CNNs.

Number of images classified				
GoogLeNet		SSc	Controls	True class
	SSc	3359 (TP)	1201 (FN)	
	Controls	1517 (FP)	10,111 (TN)	

	Predicted class			
EfficientNetB0	SSc	3666	894	True class
	Controls	989	10,639	
	Predicted class			

TP, true positive; TN, true negative; FP, false positive; FN, false negative.

**TABLE 3:** Combined confusion matrix of participant-based classification for GoogLeNet and EfficientNetB0 CNNs.

Numbers of participants classified				
GoogLeNet	Number of participants	SSc	Controls	True class
	SSc	15	5	
	Controls	7	44	

	Predicted class			
EfficientNetB0	SSc	16	4	True class
	Controls	5	46	
	Predicted class			

**TABLE 4:** Performance of image-based classification and participant-based classification for GoogLeNet in terms of diagnostic test accuracy, sensitivity and specificity (95% CI range estimates shown).

GoogLeNet CNN model diagnostic test performance (%) along with their 95% CI ranges		
	Image-based	Participant-based
Accuracy (%)	83.2 (82.6–83.8)	83.1 (72.3–90.9)
Sensitivity (%)	73.7 (72.4–74.9)	75.0 (50.9–91.3)
Specificity (%)	86.9 (86.3–87.6)	86.3 (73.7–94.3)

**TABLE 5:** Performance of image-based classification and participant-based classification for EfficientNetB0 in terms of diagnostic test accuracy, sensitivity and specificity (95% CI range estimates shown).

EfficientNetB0 CNN model diagnostic test performance (%) along with their 95% CI ranges		
	Image-based	Participant-based
Accuracy (%)	88.4 (87.8–88.8)	87.3 (77.2–94.0)
Sensitivity (%)	80.4 (79.2–81.5)	80.0 (56.3–94.3)
Specificity (%)	91.5 (90.9–91.9)	90.1 (78.6–96.7)

DL was also compared against traditional ML T-F wavelet classification using the KNN and LDA classifiers. The results of the T-F ML analysis in terms of diagnostic test accuracy, sensitivity and specificity are shown in Table 6. Using the LDA classifier the diagnostic test accuracy obtained for subject based classification was 66.2 (53.9–77.0) %, sensitivity 65.0 (40.8–84.6) % and specificity 66.7 (52.1–79.2) %. Using the KNN classifier the diagnostic test accuracy obtained for subject based classification was 76.1 (64.5–85.4) %, sensitivity 40.0 (19.1–63.9) and specificity 90.2 (78.6–96.7) %. The 95% CI range for accuracy, sensitivity and specificity for participant-based classification in DL was 72.3%–94.0%, 50.9%–94.3% and 73.7%–96.7% respectively, whereas the corresponding performance range for the ML experiment was 53.9%–85.4%, 19.1%–84.6%, and 52.1%–96.7% respectively.

**TABLE 6:** Performance of LDA and KNN comparator machine learning classifiers on a participant-basis in terms of diagnostic test accuracy, sensitivity and specificity (95% CI range estimates shown).

Machine learning (ML) model diagnostic test performance (%) along with their 95% CI ranges for participant-based classification		
	LDA	KNN
Accuracy (%)	66.2 (53.9–77.0)	76.1 (64.5–85.4)
Sensitivity (%)	65.0 (40.8–84.6)	40.0 (19.1–63.9)
Specificity (%)	66.7 (52.1–79.2)	90.2 (78.6–96.7)

## 4 Discussion

**FIGURE 8:**
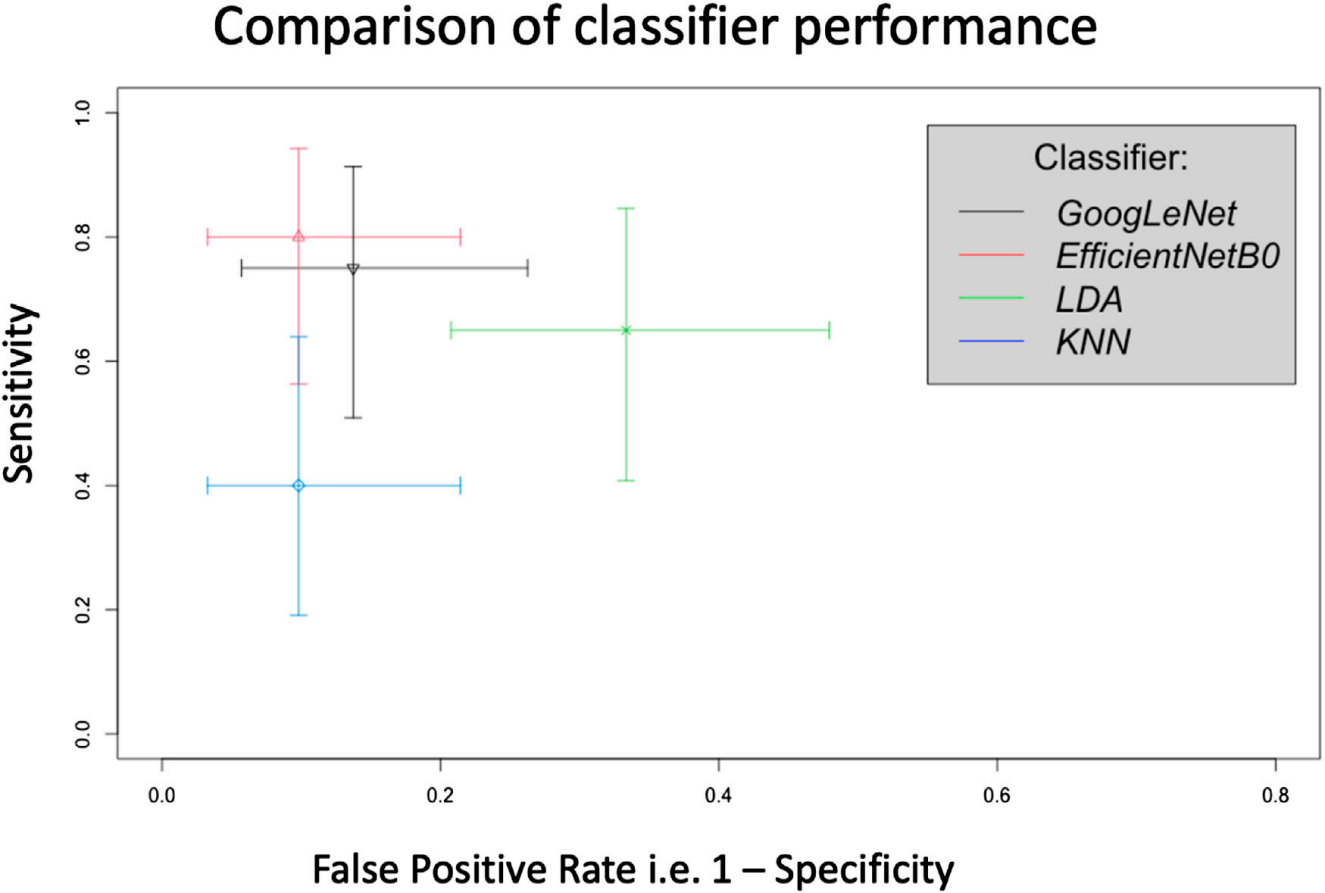
Crosshair plot giving a comparison of classifier performance using test sensitivity *versus* false positive rate (i.e., 1-specificity) for SSc *versus* Control on a participant basis. In this representation a sensitivity of 1 = 100% and similarly for the false positive rate.

This pilot study has shown that AI analysis, i.e., using 2 different types of deep learning classifier, can differentiate between the PPG recordings from Controls and SSc on a participant-basis and give approximate test accuracies of 83% (for GoogLeNet, released circa 2014) and 87% (for EfficientNetB0, released circa 2019). Figure 8 shows crosshair plot showing the comparison of performance of the four classifiers used. The overall test performance of EfficientNetB0 is marginally better overall than GoogLeNet but both CNNs were clearly better than the conventional ML classification approaches (i.e., LDA and KNN, accuracies were only 66% and 76%, respectively).

Previous published research studies on SSc diagnostics using PPG data, particularly Rosato et al. (2010), Rosato et al. (2011), McKay et al. (2014) and Mamontov et al. (2020), had each relied on statistical and manual analysis approaches to differentiate between subject groups. Manual analysis limits the ability of these techniques to be implemented in practical clinical test settings and for larger datasets. To the best of the authors’ knowledge (literature search made to January 2023), there are no studies on contact-based PPG measurements for the study of SSc using DL analytics. In our work we also aimed to fill in the gaps found in the earlier SSc PPG literature. SSc is also a growing area of interest using deep learning and skin histology type imaging, for example, the recent pilot study by Akay et al., 2021.

**Advantages with our measurement and analysis approach:** This DL-PPG proof-of-concept study presents a straightforward and effective method using deep learning as compared to conventional ML and statistical analysis approaches of differentiating between subject groups. The transfer learning ability of CNNs previously trained on thousands to millions of non-medical images allows quicker retraining on the disease specific dataset, thereby saving time and computational cost. DL eliminates the need to collect an ECG signal to give a cardiac timing reference as in conventional analysis of the PPG features on a beat-by-beat basis. CNNs can learn hundreds of features automatically from the data, thereby eliminating the need to extract key PPG features by domain experts.

Two different CNN architectures, namely, GoogLeNet and EfficientNetB0, have been employed in this research as they both have been trained on the same ImageNet database (Krizhevsky et al., 2017) and have same input dimension of 224 × 224 × 3. However, the 2 structures are different with GoogLeNet having 144 layers and approximately 6.9 million parameters (Tan and Le, 2019) and EfficientNetB0 having 290 layers but a smaller number (i.e., ∼5.3 million) parameters. The initial layers of a CNN learn low level features from the input images such as edges whilst the deeper level layers learn advanced features of input images such as constituent parts (LeCun et al., 2015). The CNN acts as a classifier too and the last layer namely, the output layer contains as many output nodes as the number of classes of data fed into the network. In this work, the 71 participants were first partitioned into 10-folds using stratified CV, wherein 9 folds of subjects are used for training the network and the remaining 1-fold of subjects were used for its testing. The participant-based division we employed ensured that the data from same subject does not fall into training and testing simultaneously which could have led to testing the same type of images as in training and hence falsely exaggerated the performance.

In this work, first T-F image-based classification of SSc from Controls was carried out. This was done because the PPG recordings were converted into a series of images and the classifiers trained using these. Then using post-processing, the classification was performed using the participants classified into SSc and Control classes, which is clinically and practically the desired case. Figure 6 shows that more recent CNN EfficientNetB0 has higher sensitivity and specificity and hence produced a better classification performance as compared to GoogLeNet. This could be due to EfficientNetB0 having more layers than other CNN GoogLeNet and hence learning more features from the input data, whilst computing faster (Tan et al., 2019). Future studies could investigate the effects of changing the network parameters to study effects on classification performance. It also shows that both DL architectures, namely, GoogLeNet and EfficientNetBo, can give higher performance in terms of sensitivity and specificity as compared to ML classifiers namely, LDA and KNN. This is because both types of CNN extract several hundreds of features from inputs thereby learning the inherent details of the data, as compared to tens of features chosen manually for the traditional ML classification techniques.

Another point to mention is the good sensitivities (in percent for participant based) of DL architectures namely, GoogLeNet having 75.0 (95% CI 50.9–91.3) % and EfficientNetB0 having 80.0 (56.3–94.3) %. These could be considered better than for the specific antibody blood tests performance summarised in Table 7 since only 10 of the 20 (50%) SSc participants were positive for either the ACA or Scl-70 autoantibodies. It is noted that there is now easier access to extended scleroderma autoantibody panels but at the time of the original PPG data collection only 2 antibody tests were available (ACA and Scl70). High accuracy is of course very important to help make this PPG-based SSc classification technique clinically relevant. Clinically because of the morbidity and mortality involved in SSc, it is particularly important to identify all such patients and therefore the sensitivity of the test should be high. Specificity should also be high as falsely labelling a person who has not got the disease could lead to unnecessary further testing (inefficient use of time and resources) as well as likely significant anxiety for the patient. The ultimate gold standard of diagnosing SSc remains the expertise of the clinicians but this work shows the potential of DL-based classification using PPG to help screen SSc patients in the future. In future this study could be carried out on a bigger dataset comprising a greater number of SSc and control participants to further validate our initial findings. The pilot work also helps in the design of advanced analysis sub-systems of PPG technology for SSc diagnostics.

**TABLE 7:** Clinical summary for the 20 SSc participants included in the study.

1	Disease subtype, number (% out of total participants)	11 diffuse cutaneous SSc (55%) 9 limited cutaneous SSc (45%)
2	Disease phase, n (%)	Early: 5 (25%)
		Intermediate/late: 13 (65%), 2 missing data
3	SSc specific autoantibodies, n (%)	Scl70 positive 4 (20%)
		ACA positive 6 (30%)
4	MRSS for the 17 sites (maximum possible score 51)	
	Median (IQR)	4 (11)
	Range	(1–33)
5	Finger digital ulcers, n (%)	6 (30%)
6	Ulcers (foot), n (%)	3 (15%)
7	Capillaroscopy abnormal, n (%)	9 (45%)
8	Thermography abnormal, n (%)	7 (35%)
9	Patients with CRP >5 mg/L, n (%)	4 (20%)
10	SHAQ, mean (range)	9 (2–22)

ACA, anticentromere antibody; MRSS, modified rodnan skin score; IQR, interquartile range, CRP = C-Reactive Protein, SHAQ, Scleroderma Health Assessment Questionnaire.

**Limitations and Future Work:** In this pilot study there were 20 SSc and 51 Control participants analysed and thus had a degree of imbalance between the classes. The groups were not fully-age-matched either although the age difference here could be considered modest in terms of vascular ageing. Future wider studies, involving more patients should explore the impact of matching across age, BMI and blood pressure. Our study was sufficient though to show the capability of the DL-PPG approach as well as highlight its opportunities and challenges. Stratified cross validation had been used to ensure equal division of minority class cross the different training folds. In this work the patient control PRP participants have been grouped with HC to form the combined Control class because of the clinical relevance of finding life threatening disease cases against the non-life-threatening control cases. The sensitivity or accuracy of classification had also not been maximised. Future studies will address these limitations to study the effect on classification performance by including a balanced study population and with greater SSc participant numbers. Noting though that SSc is a rare disease and it is not straightforward to recruit patients and collect very large data sets except perhaps across multiple clinical centres specialising in the condition. (Mitchell, 2006; Fagerland, 2012; Goodfellow et al., 2016; Allen et al., 2020; Huthart et al., 2020; Dong et al., 2021; Kyriacou and Allen, 2021; Phillips, 2022; Iqbal, 2023).

## 5 Summary

We have demonstrated in this first proof-of-concept study that GoogLeNet and EfficientNetB0 DL-PPG analytics can detect SSc with an accuracy of 83.1 (95% CIs 72.3–90.9) % and 87.3 (77.2–94.0) % for participant-based classification, respectively. The results from our novel DL-PPG classification technique appear better than for conventional ML methods. The DL-PPG sensitivity (75.0% and 80.0% for GoogLeNet and EfficientNetB0, respectively) is clearly better than for the standard immunological biomarkers for SSc available at the time our original PPG data collection for the research. DL-PPG has shown promise and should be developed further to become an accessible test for the benefit of patients with Systemic Sclerosis as well as for those with Raynaud’s.

## Data Availability

The data analyzed in this study is subject to the following licenses/restrictions: The datasets presented in this article are not readily available because the participants involved at the time of the original data collection did not consent to their PPG measurements being shared openly such as in a public repository. Requests to access these datasets should be directed to Not applicable.
